# Symptom burden and life challenges reported by adult chordoma patients and their caregivers

**DOI:** 10.1007/s11136-017-1544-2

**Published:** 2017-03-17

**Authors:** Paula H. Song, Hadi Beyhaghi, Josh Sommer, Antonia V. Bennett

**Affiliations:** 1 Department of Health Policy and Management, The University of North Carolina at Chapel Hill, 1105A McGavran-Greenberg Hall, CB #7411, Chapel Hill, NC 27599-7411 USA; 2 Chordoma Foundation, Durham, NC USA

**Keywords:** Chordoma, Symptom burden, Patient-reported outcomes, Quality of life

## Abstract

**Purpose:**

This study aims to characterize the symptom burden and life challenges that chordoma patients and their caregivers experience.

**Methods:**

In this cross-sectional study, we analyzed data from the Chordoma Foundation online community survey conducted in 2014. Frequency counts and percentages were calculated to determine the prevalence of self-reported symptoms and life challenges in the sample. We used Fisher’s exact test to compare self-reported symptoms among subgroups with different disease status, tumor locations, and treatments received.

**Results:**

Among the survey participants, 358 identified themselves as chordoma patients and 202 as caregivers. The majority of the patients were over 45 years (72%), male (56%), educated beyond high school degree (87%), and from North America (77%). Skull base was the most prevalent tumor location (40%). Chronic pain (35%) was the most commonly reported symptom followed by depression or severe anxiety (32%) and chronic fatigue (31%). Among patients, the most commonly reported challenges included delayed care (37%), long-term disability (33%), and confusion or unanswered questions about chordoma (28%). For caregivers, grief (55%), delayed diagnosis (47%), and difficulty helping the patient cope with his or her disease (45%) were most common.

**Conclusions:**

Our study findings suggest a high symptom burden and life challenges among chordoma patients and their caregivers. This study provides preliminary, limited estimates of the prevalence of a wide range of self-reported symptoms and challenges that will inform the assessment of patient-reported outcomes in future clinical trials and help clinicians better manage chordoma patients’ symptoms.

**Electronic supplementary material:**

The online version of this article (doi:10.1007/s11136-017-1544-2) contains supplementary material, which is available to authorized users.

## Introduction

Chordoma is a rare bone cancer that accounts for 1–4% of all bone malignancies [1]. Chordoma is a highly morbid and potentially fatal disease that involves complex medical decisions, and the low incidence of chordoma creates challenges for tailoring care. Chordoma tumors are thought to be derived from notochord remnants that may persist anywhere along the axial skeleton [1, 2]. Chordomas are generally slow growing, chemotherapy-, and radiotherapy-resistant tumors that are locally invasive [1].

Population-based studies using the Surveillance, Epidemiology, and End Results (SEER) database estimate the age-adjusted incidence of all chordoma cases to be 0·08 to 0.089 per 100,000. Chordoma is more common in men and the incidence is highest among 50–60 years of age [1–4]. Chordoma is less frequent among patients younger than 40 years and rarely affects children and adolescents (less than 5% of all chordoma cases) [1]. The anatomic distribution of chordomas is almost equal among the skull base (32%), mobile spine (33%), and sacrum (29%) [1] Chordoma accounts for over 50% of primary sacral tumors [1] and less than 1% of all intracranial neoplasms [2].

Most studies estimate the survival of chordoma patients using data from the SEER database, showing a median overall survival of 7.8 years [4]. Surgery plus radiation therapy is estimated to increase the overall median survival time to 9.2 years. Age and treatment modality are significant determinants of patients' survival [3]. Younger patients (aged <40 years) survive longer compared with older patients (10-year relative survival of 68% vs. 43%) [4]. Specifically, age >50 years is associated with an increase in mortality rate. The estimated age-standardized 5-year, 10-year, and 20-year survival rates for patients diagnosed between 1973 and 2009 are 72, 48, and 31%, respectively [4]. Improved survival is seen over time with 5-year survival rates of 48, 73, and 81% for the 1975–1984, 1985–1994, and 1995–2004 cohorts, respectively [3]. Primary tumor site has not shown to influence chordoma patients’ survival [5, 6].

As chordoma patients survive longer, the importance of identifying determinants of health-related quality of life (HRQOL) in guiding comprehensive patient care increases [2]. Chordoma progresses slowly but continuously, affecting multiple vital functions, which results in disability and death. As chordoma progresses, it affects patients’ physical, social, and mental wellbeing and increases the need for caregiver support [2]. However, symptom burden and other determinants of HRQOL remain poorly defined in patients with chordoma [2]. Improving HRQOL during and after treatment is a primary endpoint underlying clinical decision-making and the development of optimal treatment regimens for patients with chordoma [7]. A better understanding of the symptom burden and main challenges that chordoma patients and caregivers face will help clinicians and palliative care providers improve the quality of care for chordoma patients.

The number of clinical trials testing different interventions for treating chordoma has significantly increased over the past few years [8]. Currently, three Phase 1, two Phase 2, and one Phase 3 randomized clinical trials are in progress that assess the efficacy and safety of surgery, radiotherapy, and medical therapy in treating chordoma patients [8]. Patient-reported outcomes (PROs), especially symptom burden and functional status, are increasingly assessed in oncology clinical trials for approval and reimbursement purposes [9]. Although challenges exist for assessing PROs for rare conditions like chordoma, many of the barriers are inherent to studying rare diseases rather than PRO measurement itself, and the common barriers and solutions have been documented [10]. Information about the prevalence of the symptoms and other determinants of HRQOL in patients undergoing treatment for chordoma is essential for planning PRO assessment in a clinical trial. This information supports the identification of the most relevant PRO endpoints and provides preliminary support of the content validity of PRO instruments [11].

This study aims to characterize the symptom burden and challenges to quality of life that chordoma patients and their caregivers experience.

## Methods

### Study design and setting

In this cross-sectional study, we analyzed data from a survey of the chordoma community conducted by the Chordoma Foundation to determine the prevalence of self-reported symptoms experienced by chordoma patients and the chordoma-related life challenges that patients and their caregivers experience. The Chordoma Foundation (CF) is a global non-profit organization whose mission is to improve the lives of those affected by chordoma and lead the search for a cure. In 2014, the CF conducted an internet-based survey of the chordoma community to better define the needs and range of experiences of its constituents. At the time of the survey, the CF’s contact database included 950 self-identified chordoma patients and 3,276 individuals identified as family members or friends of patients. In March 2014, a link to the survey was emailed to all members of the foundation’s database, including both patients and caregivers. The survey was open to respondents until the end of June 2014.

### Survey instrument

The domains of the survey include self-reported symptoms, chordoma-related life challenges, quality of care, and the role of the CF in serving the chordoma community worldwide. The survey, written in English, contains 35 questions and takes approximately 19 minutes to complete. The CF developed the survey with input from members of the chordoma patient community, the moderators of the chordoma survivors Facebook group, and physicians who treat chordoma patients. The survey was constructed and administered through Survey Monkey with branching logic directing patients and caregivers to answer questions relevant to their respective experiences with chordoma. The survey questionnaire is available in the electronic supplementary material.

### Statistical analysis

Due to a very small sample size of children respondents, the study analyses focus on respondents who were 18 years and older. Caregivers were defined as parents, spouse, or family members providing care to a patient with chordoma. The analysis of self-reported symptoms was limited to patient respondents because patients are the most reliable source of information regarding their symptoms [12]. The analysis of chordoma-related life challenges included data from both patient and caregiver respondents. Two separate questions with slightly different response options were included in the survey for patients and caregivers about their experience with challenges that they face as a result of chordoma (patients) and caring for a patient with chordoma (caregivers).

Frequency counts and percentages were calculated to determine the prevalence of self-reported symptoms in the sample. Fisher’s exact test was used to compare the prevalence of a wide range of symptoms (Tables 3, 4, 5) in different subgroups. Associations were calculated between the most common symptoms reported and the following clinical characteristics: location of tumor, disease status, and type of treatment received. The prevalence of chordoma-related symptoms among subgroups with different tumor locations, disease status, and type of treatment received was tabulated. Data were analyzed using Stata software version 12 (StataCorp, College Station, TX), and p < 0.05 was considered statistically significant.

## Results

### Study population

Between March and June 2014, 709 participants including chordoma patients (358), their family members (294), and friends and others (57) responded to the online CF community survey (Table 1). Among family members, i.e., parents (31%), spouses (36%), and other family members (33%), 202 participants identified themselves as a caregiver to a chordoma patient. The majority of the patient respondents were over 45 years (72%), male (56%), educated beyond high school degree (87%), and from North America (77%). Caregivers were predominantly over 45 years (62%), female (66%), educated beyond high school degree (86%), and from North America (74%). The skull base was the most common tumor location in our patient sample (40%) followed by sacrococcygeal tumors (31%) and mobile spine, i.e., neck, mid back, and lower back (27%). The majority of patients reported either disease-free status, i.e., no evidence of tumor (41%) or stable local disease (28%). The majority of patients reported receiving multiple therapies, i.e., a combination of surgery, radiotherapy, or medical therapy (57%) followed by surgery only (25%) and radiotherapy only (7%). Table 2 shows the distribution of treatment types based on the anatomic site of chordoma tumor reported by chordoma patients.

**Table 1:** Demographic characteristics of the chordoma patients and caregivers

Age	Patients (*n* = 358)		Caregivers (*n* = 202)^a^	
	No	%	No	%
18–24	16	4	12	6
25–34	31	9	27	13
35–44	54	15	36	18
45–54	84	23	41	20
55–64	105	29	52	26
65–74	52	15	26	13
74 or older	15	4	7	3
Gender				
Male	199	56	68	34
Female	157	44	133	66
Education				
Some high school or less	8	2	8	4
High school graduate or GED	36	10	19	9
Vocational college or some college	99	28	35	17
College degree	111	31	73	36
Professional or graduate degree	103	29	65	32
Region				
North America	275	77	149	74
Europe	59	16	32	16
South America	7	2	5	3
Asia	8	2	9	5
Australia and New Zealand	6	2	5	3
Africa	3	1	2	1.0
Tumor location				
Skull base	144	40	97	48
Mobile spine	97	27	58	29
Sacrum or Coccyx	111	31	42	21
Others^b^	4	1	5	2
Current status of disease				
Disease-free (no evidence of tumor)	145	41	38	19
Stable local disease	101	28	43	21
Progressive local disease	21	6	23	11
Stable metastatic disease	17	5	7	3
Progressive metastatic disease	25	7	17	8
Not sure	28	8	7	3
Treatment received				
Surgery only	89	25	25	12
Radiotherapy only	24	7	8	4
Medical therapy only	3	1	0	0
Multiple treatments	204	57	146	72
No treatment reported	10	3	5	2

Not reported: age (patient: 1, caregiver: 1); gender (p: 2, c: 1); education (p: 1, c: 2); tumor location (p: 2, c: 5); current status of disease (p: 21, c: 67); treatment received (p: 28, c: 18)

^a^ Reflects caregivers’ demographic characteristics (age, gender, education, and region) and patients’ tumor location, current status of the disease, and treatment received reported by their caregiver

^b^ Includes extra-axial, multifocal, or unknown

**Table 2:** Distribution of treatment types based on the anatomic site of chordoma tumor reported by chordoma patients (*n* = 329)

Tumor location	All	Treatment type				
		Surgery only (*n* = 88)	Radiotherapy only (*n* = 24)	Medical treatment only (*n* = 3)	No treatment (*n* = 10)	Multiple treatments^a^ (*n* = 204)
Skull base	134 (56%)	22 (16%)	11 (8%)	0 (0%)	6 (4%)	95 (71%)
Mobile spine	91 (28%)	22 (24%)	7 (8%)	1 (1%)	2 (2%)	59 (65%)
Sacrum or Coccyx	100 (30%)	42 (42%)	6 (6%)	2 (2%)	2 (2%)	48 (48%)
Other	4 (1%)	2 (50%)	0 (0%)	0 (0%)	0 (0%)	2 (50%)

Fisher’s exact test *p* = 0.004

^a^ At least two treatment types from surgery, radiotherapy, or medical treatment categories

### Patient-reported symptoms

The most common symptoms that chordoma patients reported include chronic pain (35%), depression or severe anxiety (32%), chronic fatigue (31%), difficulty walking (28%), and balance impairment (26%). The majority of patients in our sample reported experiencing at least one symptom (93%), while more than 36% experience 5 or more symptoms as a result of suffering from chordoma. Thirty patients (8%) did not respond to the question about symptoms.

The majority of self-reported symptoms were significantly associated with the location of tumor (Table 3, last column). Among patients with skull base tumors (40%), the three most common symptoms were double vision (50%), depression or severe anxiety (32%), and chronic sinus problems (31%). Among patients with sacral tumors (20%), difficulty sitting (55%), difficulty walking (53%), and sexual dysfunction (49%) were the most common, and among patients with lower back tumor (11%), the most common symptoms were chronic pain (54%), difficulty walking (49%), and limited mobility (41%). Several common symptoms were significantly different in their prevalence across different types of treatment received and disease status (Tables 4, 5).

**Table 3:** Distribution of the patient-reported symptoms associated with chordoma by tumor location

Symptom		All (*n* = 327) (%)	Chordoma tumor location				
			Skull base (*n* = 131) (%)	Mobile spine^a^ (*n* = 92) (%)	Sacrum or Coccyx (*n* = 101) (%)	Other^b^ (*n* = 3) (%)	P-value^c^
Chronic pain	(*n* = 123)	38	14	57	51	33	0.001
Depression or severe anxiety	(*n* = 113)	35	35	37	31	67	0.489
Chronic fatigue	(*n* = 110)	34	30	39	33	67	0.269
Difficulty walking	(*n* = 100)	31	7	40	54	67	0.001
Balance impairment	(*n* = 94)	29	33	26	25	67	0.205
Difficulty sitting	(*n* = 85)	26	2	20	62	33	0.001
Double vision	(*n* = 76)	23	56	1	2	0	0.001
Sexual dysfunction	(*n* = 74)	23	15	10	46	0	0.001
Limited mobility	(*n* = 63)	19	3	33	29	0	0.001
Urinary incontinence	(*n* = 52)	16	2	2	48	0	0.001
Chronic sinus problems	(*n* = 46)	14	34	1	0	0	0.001
Urinary retention	(*n* = 46)	14	1	3	42	0	0.001
Hearing loss	(*n* = 44)	13	32	1	1	0	0.001
Fecal incontinence	(*n* = 41)	13	0	1	40	0	0.001
Bowel obstruction	(*n* = 38)	12	2	7	30	0	0.001
Other vision problems	(*n* = 28)	9	16	3	4	0	0.002

Symptoms were asked using the following survey question: “Which of the following health effects have you ever suffered as a result of chordoma?”

^a^ Includes neck, mid back, and lower back

^b^ Includes extra-axial, multifocal, or unknown

^c^ P-values were calculated using Fisher’s exact test

**Table 4:** Distribution of the patient-reported symptoms associated with chordoma by treatment received

Symptom		All (*n* = 322) (%)	Treatment received					P-value^a^
			Surgery only (*n* = 87) (%)	Radiotherapy only (n = 22) (%)	Medical therapy only (*n* = 3) (%)	No treatment (*n* = 8) (%)	Multiple therapy (*n* = 202) (%)	
Chronic pain	(*n* = 122)	38	49	27	33	38	34	0.107
Depression or severe anxiety	(*n* = 111)	34	38	27	67	38	33	0.632
Chronic fatigue	(*n* = 106)	33	29	18	33	38	36	0.375
Difficulty walking	(*n* = 97)	30	34	27	33	50	28	0.502
Balance impairment	(*n* = 92)	29	22	23	33	38	32	0.394
Difficulty sitting	(*n* = 82)	25	44	18	33	50	17	0.001
Double vision	(*n* = 75)	23	13	32	33	25	27	0.046
Sexual dysfunction	(*n* = 72)	22	26	27	33	13	20	0.607
Limited mobility	(*n* = 62)	19	26	5	67	25	17	0.020
Urinary incontinence	(*n* = 49)	15	15	9	67	38	14	0.059
Urinary retention	(*n* = 46)	14	16	0	33	38	14	0.040
Chronic sinus problems	(*n* = 45)	14	5	18	0	13	18	0.022
Hearing loss	(*n* = 44)	14	6	18	33	13	16	0.050
Fecal incontinence	(*n* = 40)	12	18	14	33	13	9	0.128
Bowel obstruction	(*n* = 38)	12	15	9	67	13	10	0.075
Other vision problems	(*n* = 25)	8	3	14	33	13	8	0.084

Symptoms were asked using the following survey question: “Which of the following health effects have you (the patient) ever suffered as a result of chordoma?”

^a^ P-values were calculated using Fisher’s exact test

**Table 5:** Distribution of the patient-reported symptoms associated with chordoma by disease status (*n* = 309)

Symptom		All (*n* = 309) (%)	Current disease status						P-value^a^
			Disease-free (*n* = 138) (%)	Stable local (*n* = 87) (%)	Progressive local (*n* = 18) (%)	Stable metastatic (*n* = 17) (%)	Progressive metastatic (*n* = 24) (%)	Not sure (*n* = 25)(%)	
Chronic pain	(*n* = 117)	38	39	29	44	41	58	36	0.157
Depression or severe anxiety	(*n* = 112)	36	37	37	39	35	33	32	0.997
Chronic fatigue	(*n* = 103)	33	30	38	17	35	50	28	0.233
Difficulty walking	(*n* = 92)	30	30	16	56	35	58	24	0.001
Balance impairment	(*n* = 90)	29	22	34	33	29	38	36	0.290
Difficulty sitting	(*n* = 76)	25	33	11	28	12	50	8	0.001
Double vision	(*n* = 72)	23	17	39	28	12	17	16	0.004
Sexual dysfunction	(*n* = 72)	23	22	14	33	53	38	20	0.006
Limited mobility	(*n* = 62)	20	22	8	28	35	42	12	0.001
Urinary incontinence	(*n* = 48)	16	20	4	17	12	38	12	0.001
Chronic sinus problems	(*n* = 44)	14	9	26	11	18	8	8	0.009
Urinary retention	(*n* = 43)	14	20	6	6	18	17	12	0.051
Hearing loss	(*n* = 41)	13	6	23	17	18	13	16	0.005
Fecal incontinence	(*n* = 39)	13	17	4	17	12	21	12	0.021
Bowel obstruction	(*n* = 36)	12	12	5	22	18	29	8	0.010
Other vision problems	(*n* = 27)	9	4	18	17	6	4	0	0.003

Symptoms were asked using the following survey question: “Which of the following health effects have you (the patient) ever suffered as a result of chordoma?”

^a^ P-values were calculated using Fisher’s exact test

### Challenges experienced by chordoma patients and caregivers

Table 6 reports the prevalence of challenges that patients and caregivers experienced as a result of chordoma in the following domains: emotional health and coping; employment and finances, access to care and information; and quality of care. Eight patients (2%) and four caregivers (2%) did not respond to the question about challenges faced as a result of suffering from chordoma. The most common challenges reported by patients were delayed care (37%), long-term disability (33%), confusion or unanswered questions about chordoma (28%), difficulty finding experienced physicians or treatment centers (27%), misdiagnosis (24%), and short-term disability (24%). Among caregivers, the most frequently reported challenges included grief (55%), delayed care (47%), difficulty helping the patient cope with his/her illness (45%), confusion or unanswered questions about chordoma (45%), difficulty finding experienced physicians or treatment centers (43%), and misdiagnosis of the patient (28%).

**Table 6:** Challenges that patients/caregivers face as a result of having/caring for a patient with chordoma

	Patients (*n* = 358)		Caregivers (*n* = 202)	
	No.	Percent	No.	Percent
Emotional health and coping				
Feelings of loneliness or isolation	104	29	87	43
Difficulty coping/helping the patient cope with his/her illness	93	26	91	45
Grief^a^	–	–	111	55
Family conflict	34	10	46	23
Difficulty helping children or other family members cope	47	13	67	33
Difficulty talking about chordoma and how it has affected my life	64	18	43	21
Employment and finances				
Change in career or reduced ability to work	118	33	63	31
Short-term disability^b^	84	24	–	–
Long-term disability^b^	119	33	–	–
Loss of employment	74	21	20	10
Financial distress (including bankruptcy or foreclosure)	59	17	39	19
Access to care and information				
Denial of insurance coverage for the patient’s recommended treatment	45	13	34	17
Loss of health insurance^b^	20	6	–	–
Inability to pay for the patient’s recommended treatment	28	8	20	10
Inability to pay for travel, lodging, or other treatment-related expenses	45	13	28	14
Confusion or unanswered questions about chordoma	101	28	90	45
Difficulty finding experienced physicians or treatment centers	96	27	86	43
Quality of care				
Delayed diagnosis of patient	134	37	95	47
Misdiagnosis of patient	85	24	56	28
Difficulty dealing with physicians or medical staff	77	22	50	25
Patient received wrong or inappropriate care	64	18	49	24

The challenges were asked in the following questions in the survey: “Which of the following challenges have you faced as a result of your experience with chordoma?” and “Which of the following challenges have you faced in caring for someone with chordoma?”

^a^ Response option only available to respondents who identify themselves as caregivers of chordoma patients

^b^ Response option only available to respondents who identify themselves as patients

## Discussion

Although several studies of chordoma symptoms and quality of life exist, they focus on a small number of cases, limited list of symptoms, or a specific tumor location or treatment modality [2, 7, 13, 14]. This study adds to the existing literature by investigating self-reported symptom burden and life challenges among a large group of chordoma patients and caregivers across different tumor characteristics. Our study data suggest high symptom burden among chordoma patients that varies based on the location of the tumor, disease status, and type of treatment received. In addition, our findings show that the chordoma patients and caregivers face many medical, emotional, and healthcare challenges. Receiving inappropriate care including but not limited to delayed diagnosis or misdiagnosis and difficulty finding experienced physicians or medical centers were among the most commonly cited challenges. Our study results provide a better understanding of the challenges associated with chordoma, with the aim of helping improve the quality of care provided to chordoma patients.

Our study also provides prevalence data for a comprehensive list of patient-reported symptoms as well as patient- and caregiver-reported life challenges associated with chordoma. These findings can inform the design of clinical trials and guide the development of chordoma-specific PRO instruments or selection of existing generic instruments. Information about the prevalence of symptoms and life challenges is necessary for identifying which concepts should be assessed. For detailed information about symptoms and life challenges specific to sacral chordoma patients and an example of using existing generic (non-disease specific) PRO instruments to develop a comprehensive questionnaire, see van Wulfften Palthe et al. (2016) [14]. Because of the nature of the chordoma—in that the impact of the disease varies by the location of the tumor(s), instruments for specific types of chordoma may be warranted, depending on the goals of the study. This could be operationalized as a series of independent tumor site-specific instruments or one instrument with tumor site-specific modules and branching logic. Further, our findings can be directly utilized in clinical practice. Information about prevalent symptoms and challenges faced by patients and caregivers may help improve patient–provider communication, inform decision-making, healthcare management, and ultimately improve health outcomes.

The major strengths of this study include its relatively large sample size of both chordoma patients and caregivers. Given the incidence and average survival of chordoma patients, the estimated prevalence is approximately 6 per million, or roughly 2,000 patients in the United States. Thus, the survey sample included more than 10% of the estimated United States chordoma patient population. In addition, the construction of the survey was patient-centered, and approximately 90% of respondents completed the entire questionnaire. However, we acknowledge the limitations associated with the online questionnaire administration. Our study sample included a high proportion of respondents with a university degree (65%), and was limited to those who have access to a computer, can read in English, and have the mental and physical capacity to complete the online questionnaire. In addition, because patients and their caregivers had access to the survey questionnaire, for questions that could be answered both by patients and caregivers, it is possible that some responses are counted both from a patient and his or her caregiver. Nonetheless, the distribution of age and tumor location in our sample is similar to that reported in the literature from the representative population-based estimates [1–3]. Although this survey included a large proportion (10%) of the US chordoma patient population, because of the sampling scheme that was employed in this study, the denominator is not clearly defined and the sample underrepresents population subgroups who may experience the challenges of chordoma differently. Therefore, the results of this study are limited preliminary estimates intended to be hypothesis generating and to inform future research.

Previous studies have estimated quality of life in specific subgroups of chordoma patients using validated, non-disease specific instruments, such as the 36-Item Short Form Health Survey (SF-36) and the European Organization for Research and Treatment of Cancer Quality of Life Core 30 (EORTC QLQ-C30) [2, 13]. However, the intent of the CF survey was not to estimate the quality of life scores for the study sample. Instead, the survey questionnaire was designed to document the prevalence of a wide range of symptoms and challenges experienced by chordoma patients and caregivers. The content was determined based on input from members of the chordoma patient community, the moderators of the chordoma survivors Facebook group, and physicians who treat chordoma patients, and the questionnaire included open-ended response fields for each response option of “other” to elicit any additional symptoms and issues. The CF survey was able to capture symptoms and challenges specific to chordoma that were not reflected in the more general quality of life measures used previously.

## Conclusion

Our study findings suggest a high symptom burden among patients with chordoma. In addition, many patients and caregivers are facing challenges related to access and quality of care and psychological health. This study provides preliminary, limited estimates of the prevalence of a wide range of self-reported symptoms and challenges that will inform the assessment of patient-reported outcomes in future clinical trials and will help clinicians and palliative care providers better understand and manage symptoms of chordoma patients.

## Electronic supplementary material

Below is the link to the electronic supplementary material.

Supplementary material 1 (PDF 379 KB)
