# Supplementary material for: Symptom burden and life challenges reported by adult chordoma patients and their caregivers
Source: Qual Life Res. 2017 Mar 17;26(8):2237–44. doi: 10.1007/s11136-017-1544-2 (PMC5509836; doi:10.1007/s11136-017-1544-2)
Supplement: Supplementary file 1 — Supplementary material 1 (PDF 379 KB) [file 11136_2017_1544_MOESM1_ESM.pdf]

Chordoma Foundation - Chordoma Community Survey

Demographics

Thank you for taking the time to complete this survey. The information that you provide will help us improve our services to the chordoma community.

Your answers will remain anonymous unless you choose to provide contact information on the last page of the survey. All responses will be treated as confidential.

Note: For questions about chordoma diagnosis and treatment, please answer from the perspective of the patient.

1. Gender

- Male
- Female

2. Age

- Under 18 years old
- 18-24 years old
- 25-34 years old
- 35-44 years old
- 45-54 years old
- 55-64 years old
- 65-74 years old
- 75 years or older

3. Education Level

- Some high school or secondary school
- High school or secondary school graduate
- Some college or university
- Trade/technical/vocational training
- College or university graduate
- Some postgraduate work
- Postgraduate degree

*4. Country:

*5. How are you affected by chordoma?

- Chordoma Patient
- Parent of Patient
- Spouse of Patient
- Family of Patient
- Friend of Patient
- None

6. Where in the body did your (the patient's) chordoma first arise? [Excluding tumors that may have metastasized (spread) to other parts of the body]

- ○ Clivus (skull base)
- ○ Cervical spine (neck)
- ○ Thoracic spine (mid back)
- ○ Lumbar spine (lower back)
- ○ Sacrum (pelvic area)
- ○ Coccyx (tail bone)
- ○ Extra-axial (not in the spine or skull)
- ○ Multi-focal (multiple locations along the spine or skull)
- ○ Not Sure

7. What is the current status of your (the patient's) disease?

- ○ Disease-free (no evidence of tumor)
- ○ Stable local disease (tumor is present but not growing at the primary site)
- ○ Progressive local disease (tumor is growing at the primary site)
- ○ Stable metastatic disease (tumor has spread beyond the primary site but is not currently growing)
- ○ Progressive metastatic disease (tumor has spread beyond the primary site and is currently growing)
- ○ Not sure

Other (please specify)

***1. Is the patient alive?**

- ○ Yes - patient is alive
- ○ No - patient is deceased

1. Date of death (MM/YYYY)

***1. Have you been a caregiver for a chordoma patient?**

- ○ Yes - I am/was a caregiver
- ○ No - I am/was not a caregiver

1. Which of the following challenges have you faced in caring for someone with chordoma? Select all that apply.

- ☐ Delayed diagnosis of patient
- ☐ Misdiagnosis of patient
- ☐ Patient received wrong or inappropriate care
- ☐ Difficulty dealing with physicians or medical staff
- ☐ Denial of insurance coverage for the patient's recommended treatment
- ☐ Inability to pay for the patient's recommended treatment
- ☐ Inability to pay for travel, lodging or other treatment-related expenses
- ☐ Financial distress (including bankruptcy or foreclosure)
- ☐ Feelings of loneliness or isolation
- ☐ Family conflict
- ☐ Confusion or unanswered questions about chordoma
- ☐ Difficulty finding experienced physicians or treatment centers
- ☐ Change in career or reduced ability to work
- ☐ Loss of employment
- ☐ Grief
- ☐ Difficulty helping the patient cope with his/her illness
- ☐ Difficulty helping children or other family members cope
- ☐ Difficulty talking about chordoma and how it has affected my life
- ☐ None
- ☐ Other (please explain)

1. Which of the following challenges have you faced as a result of your experience with chordoma? Select all that apply.

- ☐ Delayed diagnosis
- ☐ Misdiagnosis
- ☐ Difficulty dealing with physicians or medical staff
- ☐ Received wrong or inappropriate care
- ☐ Denial of insurance coverage for recommended treatment
- ☐ Inability to pay for recommended treatment
- ☐ Inability to pay for travel, lodging or other treatment-related expenses
- ☐ Other (please explain)
- ☐ Financial distress (including bankruptcy or foreclosure)
- ☐ Feelings of loneliness or isolation
- ☐ Family conflict
- ☐ Confusion or unanswered questions about chordoma
- ☐ Difficulty finding experienced physicians or treatment centers
- ☐ Short-term disability
- ☐ Long-term disability
- ☐ Change in career or reduced ability to work
- ☐ Loss of employment
- ☐ Loss of health insurance
- ☐ Difficulty coping with my illness
- ☐ Difficulty helping children or other family members cope
- ☐ Difficulty talking about chordoma and how it has affected my life
- ☐ None

Experience with Chordoma

Please tell us more about your (the patient's) experience with chordoma.

Note: Family members, please answer all questions from the patient's perspective.

Chordoma Foundation - Chordoma Community Survey

1. What symptoms or situation led to the discovery of your (the patient's) tumor? Select all that apply.

- ☐ Back pain
- ☐ Balance difficulty
- ☐ Bowel obstruction
- ☐ Discovered incidentally following an injury or accident
- ☐ Discovered incidentally through treatment for another condition
- ☐ Difficulty breathing
- ☐ Difficulty sitting
- ☐ Difficulty swallowing or speaking
- ☐ Difficulty walking
- ☐ Double vision
- ☐ Facial drooping or facial nerve paralysis
- ☐ Fainting
- ☐ Fecal incontinence
- ☐ Gastrointestinal changes
- ☐ Neck pain
- ☐ Numbness or tingling
- ☐ Other changes in vision
- ☐ Other Pain
- ☐ Sexual dysfunction
- ☐ Stiffness
- ☐ Urinary retention
- ☐ Urinary incontinence
- ☐ Other (please explain)

2. When did you (the patient) first experience the onset of these symptoms? (MM/YYYY)

3. Before your (the patient's) tumor was discovered, what else, if anything, were you told could be causing these symptoms?

4. When was your (the patient's) tumor first discovered? (MM/YYYY)

5. Once a tumor was discovered and before the diagnosis was confirmed, what else were you (the patient) told the tumor could possibly be besides chordoma? Select all that apply.

- ☐ Benign bone tumor
- ☐ Benign notochordal cell tumor
- ☐ Bone cancer or tumor
- ☐ Brain cancer or tumor
- ☐ Chondrosarcoma
- ☐ Congenital defect
- ☐ Cyst
- ☐ Epidermoid
- ☐ Infection
- ☐ Meningioma
- ☐ Metastatic cancer
- ☐ Notochordal rest
- ☐ Stress fracture
- ☐ Other (please explain)

6. Was the first definitive diagnosis you (the patient) received the correct diagnosis of chordoma?

- ○ Yes
- ○ No

If no, what wrong diagnosis did you receive?

7. When did you (the patient) receive a confirmed diagnosis of chordoma? (MM/YYYY)

8. What treatments have you (the patient) had? Select all that apply.

- [ ] Carbon ion therapy
- [ ] Chemotherapy
- [ ] Immune therapy (including a cancer vaccine or immune checkpoint inhibitor)
- [ ] Intensity modulated radiotherapy (IMRT)
- [ ] Proton beam radiation
- [ ] Surgery
- [ ] Stereotactic Radiosurgery (including CyberKnife and Gamma Knife)
- [ ] Targeted therapy (including drugs such as Gleevec, Tarceva, Avastin, etc.)
- [ ] Have not had treatment

Other (please explain)

9. At which hospitals have you (the patient) been treated?

Hospital 1

Hospital 2

Hospital 3

Hospital 4

Hospital 5

10. Which of the following health effects have you (the patient) ever suffered as a result of chordoma? Select all that apply.

- [ ] Balance impairment
- [ ] Blindness
- [ ] Bone fracture
- [ ] Bowel obstruction
- [ ] Chronic fatigue
- [ ] Chronic infection
- [ ] Chronic pain
- [ ] Chronic sinus problems
- [ ] Cognitive impairment
- [ ] Colostomy
- [ ] Depression or severe anxiety
- [ ] Difficulty sitting
- [ ] Difficulty walking
- [ ] Double vision
- [ ] Facial paralysis
- [ ] Fecal incontinence
- [ ] Hearing loss
- [ ] Limb paralysis
- [ ] Limb amputation
- [ ] Limited mobility
- [ ] Other vision problems
- [ ] Radionecrosis
- [ ] Sexual dysfunction
- [ ] Speech impediment
- [ ] Spinal fluid leak
- [ ] Urinary retention
- [ ] Urinary incontinence
- [ ] None
- [ ] Other (please explain)

11. Has anyone else in your family ever been diagnosed with chordoma?

- ( ) Yes
- ( ) No

1. Which of your relatives have been diagnosed with chordoma?

- [ ] Parent
- [ ] Child
- [ ] Sibling
- [ ] Grandparent
- [ ] Aunt/Uncle
- [ ] Cousin
- [ ] Distant Relative
- [ ] Other (please explain)

Interaction with the Chordoma Foundation

Please tell us about your interactions with the Chordoma Foundation

1. How did you first hear about the Chordoma Foundation?

- [ ] Chordoma Foundation brochure in a clinic or hospital
- [ ] Chordoma Foundation Facebook page
- [ ] Chordoma Survivors Facebook Group
- [ ] Facebook advertisement
- [ ] Friend or family member
- [ ] Google advertisement
- [ ] Healthcare provider
- [ ] Link from another website
- [ ] News story
- [ ] Online Chordoma Survivors Support Group
- [ ] Search engine
- [ ] Other (please explain)

2. Where were you (the patient) in your chordoma journey when you first heard about the Chordoma Foundation?

- Before onset of symptoms
- After onset of symptoms but before discovery of tumor
- After discovery of tumor but before being treated
- After first treatment
- After a recurrence was discovered
- I don't know where the patient was in his or her progression

3. In what ways has the Chordoma Foundation helped you? Please rank them from very helpful (1) to not at all helpful (5).

	1 (Very Helpful)	2	3	4	5 (Not Helpful)	N/A
Gave me an opportunity to contribute my (the patient's) tissue to research	○	○	○	○	○	○
Gives me a sense of hope	○	○	○	○	○	○
Gives me a sense of purpose	○	○	○	○	○	○
Gives me a way to show support for a friend or loved one	○	○	○	○	○	○
Helped me (the patient) avoid health problems	○	○	○	○	○	○
Helped me connect with others in the chordoma community	○	○	○	○	○	○
Helped me get emotional support	○	○	○	○	○	○
Helped me (the patient) enroll in a clinical trial	○	○	○	○	○	○
Helped me find useful resources or information	○	○	○	○	○	○
Helped me (the patient) find experienced physicians	○	○	○	○	○	○
Helped me (the patient) get a diagnosis	○	○	○	○	○	○
Helped me (the patient) obtain better care	○	○	○	○	○	○
Helped me understand chordoma	○	○	○	○	○	○
Helped me understand treatment options	○	○	○	○	○	○
Keeps me informed about new research and treatment options	○	○	○	○	○	○

4. What information on the Chordoma Foundation's website did you find useful? Select all that apply.

- ☐ About chordoma
- ☐ Chordoma Foundation research programs
- ☐ Clinical trials list
- ☐ Doctor directory
- ☐ Donating to the Foundation
- ☐ Getting support
- ☐ Helpful resources page
- ☐ How to connect with others in the chordoma community
- ☐ How to contribute tumor tissue for research
- ☐ How to raise money for the Foundation
- ☐ Latest updates
- ☐ Personalized oncology service providers list
- ☐ Radiation therapy centers list
- ☐ Stories about other patients
- ☐ Treatment options
- ☐ The website was not helpful
- ☐ Other (please specify)

Improving Services to the Community

Help us learn how we can improve our services to chordoma patients, family, and friends.

1. What additional information should the Chordoma Foundation provide to the community?

2. What additional resources or services should the Chordoma Foundation provide to the community?

3. What information or advice do you wish you had received when you (or your friend/family member) were first diagnosed?

4. Where else have you found trusted information about chordoma?

Information Source 1

Information Source 2

Information Source 3

Information Source 4

Information Source 5

Getting involved

Your involvement is important to our efforts to serve the chordoma community and advance research to improve the treatment of chordoma. We'd like to know how we can best work with you to accomplish our shared mission.

1. What motivates or inspires you to support this cause? Select all that apply.

- ☐ I want to help find a cure for myself
- ☐ I want to help find a cure for a friend or loved one
- ☐ I have been served by the Foundation and I want to give back
- ☐ I want to prevent others from facing the challenges that I have faced
- ☐ I want to help make sure that all chordoma patients get access to the best treatment possible
- ☐ I am impressed with the progress the Foundation has made
- ☐ I am impressed with the Foundation's approach to advancing research
- ☐ I am inspired by the Foundation's team and/or story
- ☐ I view the Foundation as a model of effective medical research that could ultimately have an impact beyond chordoma
- ☐ I lost a loved one to chordoma and choose to honor their legacy through the Foundation
- ☐ I am close to someone affected by chordoma and show my care for them by supporting the Foundation
- ☐ Other (please explain)

2. In which ways would you be interested in supporting this cause? Select all that apply.

- [ ] Contribute tissue to the Foundation's Biobank
- [ ] Donate cash to the Foundation (including via check or credit card)
- [ ] Donate stock or other property to the Foundation
- [ ] Donate goods or professional services from my company
- [ ] Encourage others to donate to the Foundation
- [ ] Help organize a Chordoma Community Conference or educational event
- [ ] Host a fundraiser for the Foundation
- [ ] Include a bequest to the Foundation in my will
- [ ] Introduce the Foundation to a potential philanthropist or other source(s) of capital
- [ ] Make a Perseverance Pledge to the Foundation (a monthly recurring donation)
- [ ] Set up a Champion's Page (personal fundraising page)
- [ ] Serve as a peer mentor for others in the chordoma community facing a similar situation
- [ ] Serve as a regional ambassador for the Foundation
- [ ] Share an Uncommon Story (a story of inspiration or hope on the Foundation's website)
- [ ] Translate/communicate in foreign languages
- [ ] Volunteer my skills (e.g. communications, social work, IT)
- [ ] Volunteer on site at an upcoming Foundation event
- [ ] Other (please specify)

3. Do you have any other comments or suggestions you would like to share with us?

Keep In Touch

We would like to keep you updated with the latest information about chordoma research and treatment. If you would like to receive regular updates, please fill in the contact information below.

1. First Name

2. Last Name

3. Contact Information

Address 1:

Address 2:

City/Town:

State/Province:

ZIP/Postal Code:

Country:

Email Address:

Phone Number:

Thank You

Thank you for completing this survey. We appreciate your feedback and look forward to using your suggestions to improve our services to chordoma patients and their families! If you have any questions, or would like to share additional information with us, please call us at 919-809-6779 or email us at info@chordoma.org

If you or your loved one has been diagnosed with chordoma, please follow the links below to learn more about the services we offer and how we can help:

Newly Diagnosed Patients: http://www.chordomafoundation.org/newly-diagnosed/

Find a Doctor: http://www.chordomafoundation.org/doctor-directory/

Clinical Trials: http://www.chordomafoundation.org/clinical-trials/

Donate Tissue: http://www.chordomafoundation.org/biobank/

Get Support: http://www.chordomafoundation.org/chordoma-support/

Helpful Resources: http://www.chordomafoundation.org/helpful-resources/
